# First report of the complete mitogenome of *Microchironomus tabarui* Sasa, 1987 (Diptera, Chironomidae) from Hebei Province, China

**DOI:** 10.1080/23802359.2021.1970638

**Published:** 2021-09-06

**Authors:** Fan-Qing Kong, Yan-Chu Zhao, Jia-Lin Chen, Xiao-Long Lin

**Affiliations:** a Ecological Environment Monitoring and Scientific Research Center of Haihe River Basin and Beihai Sea Area, Ministry of Ecological Environment, Tianjin, China; b College of Life Sciences, Nankai University, Tianjin, China

**Keywords:** Chironomids, bioindicator, mitochondrial genome, phylogeny

## Abstract

*Microchironomus tabarui* Sasa, 1987 is an important bioindicator for freshwater ecosystem monitoring. Although COI barcode analyes have been performed on *M*. *tabarui*, the mitogenome of this taxon has not been assembled and analyzed. Here the complete mitogenome of *M*. *tabarui* was sequenced and analyzed to confirm the systematic and phylogenetic history of this species. The mitogenome is 15,667 bp long with high A + T content and consists of 13 protein-coding genes, 22 tRNA genes, two rRNA genes, and a noncoding control region. The phylogenomic analysis support monophyletic Chironominae and close relationship between *M*. *tabarui* and *Chironomus*. Our results indicate that mitogenomes showed strong signals in phylogenetic reconstructions at the genus level of Chironominae.

*Microchironomus tabarui* Sasa, 1987 is classified in the subfamily Chironominae, Chironomidae, one of the most diverse group of aquatic Diptera with around 6300 described species (P. Ashe, personal communication). Due to their high species diversity and ability to inhabit different types of water bodies, chironomid larvae are important bioindicators for freshwater ecosystem monitoring (Ferrington 2008). Due to variable morphological features, entomologists face great challenges in identifying chironomids. Mitogenomic data have been broadly used in molecular identification and phylogenetic studies of Diptera (e.g., Yan et al. 2019; Miao et al. 2020; Li et al. 2020). However, only a few Chironomidae mitogenomes have been deciphered (Beckenbach 2012; Kim et al. 2016; Deviatiiarov et al. 2017; Lei et al. 2021; Zheng et al. 2021). In this study, we provide the first complete mitochondrial genome of *M. tabarui*.

The adult male of *M. tabarui* was collected from Hengshui, Hebei, China (37.651626°N, 115.650831°E) on 1 September 2020. Total genomic DNA was extracted from the muscle tissues of the head and thorax of an adult using the DNeasy Blood and Tissue kit (QIAGEN Sciences, Valencia, CA, USA). The DNA and voucher specimen of *M. tabarui* is deposited in the College of Life Sciences, Nankai University, Tianjin, China (https://sky.nankai.edu.cn, Xiao-Long Lin, lin880224@gmail.com) under the voucher number XL3993. COI of *M. tabarui* (GenBank accession: KJ188136) was used as bait to iterate and assemble the mitogenome of *M. tabarui*. The genomic DNA was subsequently pooled with other insect species and sequenced using the Illumina Nova6000 (PE150, Illumina, San Diego, CA) platform with an insert size of 350-bp and a paired-end 150-bp sequencing strategy at Novogene Co., Ltd. (Beijing, China). Four Gb clean data were obtained from the library after trimming using Trimmomatic (Bolger et al. 2014). The software IDBA-1.1.1 (Peng et al. 2012) was employed to assemble the data with similarity set to be 0.98. The mitogenome of *M. tabarui* was then identified using a Blast search (Altschul et al. 1990) with COI as the bait sequence (Crampton-Platt et al. 2015), and the percentage of identical matches was 100%. The mitogenome annotation was conducted following Zheng et al. (2020).

The mitogenome of *M. tabarui* is 15,667 bp in length (GenBank accession No. MZ261913), containing 13 protein-coding genes (PCGs), two ribosomal RNA genes, 22 transfer RNA genes, and one noncoding control region. The overall nucleotide composition was 39.8% of A, 36.6% of T, 14.2% of C, 9.4% of G, 76.4% of A + T content. Most of the 13 PCGs used ATN as the start codon (ATC for ATP8; ATG for ATP6, COII, COIII, CytB, ND4 and ND4L; ATT for ND2, ND3 and ND6; GTG for ND5; TTG for COI and ND1). The stop codon TAA is assigned to the 13 PCGs. Gene arrangement of the 13 PCGs is identical to that of other known Chironomidae mitogenomes. Nucleotide composition of *M*. *tabarui* is similar with other known Chironomidae mitogenomes, with a high A + T bias.

Ten mitochondrial genomes of Chironomidae and two of Ceratopogonidae available from GenBank were mined for the phylogenetic analysis. The sequences were concatenated with alignments of 13 PCGs using the default settings in MAFFT (Katoh and Standley 2013). The maximum-likelihood (ML) reconstruction was performed using IQ-TREE (Nguyen et al. 2015) with 1000 bootstraps replicates and thePMSF acid substitution model. In addition, *Culicoides arakawae and Forcipomyia makanensis* were designated as the outgroups. The result (Figure 1) clearly shows that Chironominae formed a monophyletic group. *Microchironomus tabarui* is sister to the genus *Chironomus* based on mitogenomics, which is concordant with morphology. This work provides molecular characterizations of *M. tabarui* and contributes to the phylogenetic analysis of Chironomidae.

**Figure 1.**
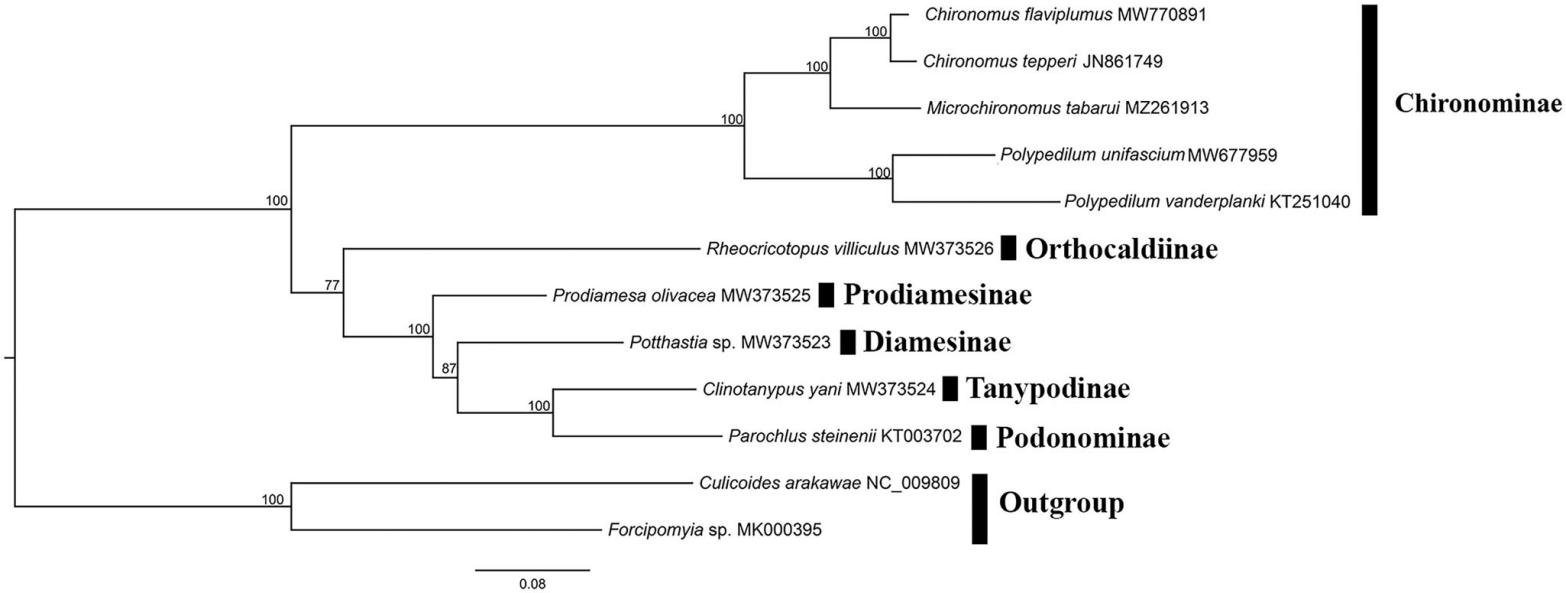
Phylogenetic tree of ten Chironomidae species based on the concatenated dataset of 13 PCGs using the maximum-likelihood (ML) method. The alphanumeric terms following species names indicate the GenBank accession numbers.

## Data Availability

The data that support the findings of this study are openly available in GenBank of NCBI at https://www.ncbi.nlm.nih.gov/ under the accession No. MZ261913. The associated BioProject, SRA, and Bio-Sample numbers are PRJNA731346, SUB9688437, SAMN19277824, respectively.
